# Radical nephroureterectomy vs kidney sparing surgery for upper tract urothelial carcinoma in solitary kidney patients: a multi-institutional analysis of the ROBUUST 2.0 registry

**DOI:** 10.1007/s00345-025-05882-0

**Published:** 2025-09-03

**Authors:** Francesco Ditonno, Alessandro Veccia, Gabriele Bignante, Zhenjie Wu, Linhui Wang, Firas Abdollah, Alex Stephens, Giuseppe Simone, Gabriele Tuderti, Randall Lee, Daniel D. Eun, Andres F. Correa, Ottavio De Cobelli, Matteo Ferro, Francesco Porpiglia, Daniele Amparore, Enrico Checcucci, Antonio Tufano, Roberto Contieri, Sisto Perdonà, Raj Bhanvadia, Vitaly Margulis, Stephan Brönimann, Nirmish Singla, James Porter, Saum Ghodoussipour, Andrea Minervini, Andrea Mari, Luca Lambertini, Alireza Ghoreifi, Omri Falik Nativ, Mark L. Gonzalgo, Daniel Sidhom, Chandru P. Sundaram, Reuben Ben-David, Ahmed Eraky, Reza Mehrazin, Takashi Yoshida, Hidefumi Kinoshita, Alireza Dehghanmanshadi, Soroush Rais-Bahrami, Margaret F. Meagher, Dhruv Puri, Ithaar H. Derweesh, Farshad S. Moghaddam, Hooman Djaladat, Riccardo Bertolo, Riccardo Autorino, Alessandro Antonelli

**Affiliations:** 1 Department of Urology, Azienda Ospedaliera Universitaria Integrata Verona, University of Verona, Verona, 37126 Italy; 2 Department of Urology, Rush University, Chicago, IL USA; 3 Department of Urology, Shanghai Changhai Hospital, Naval Medical University, Shanghai, China; 4 Henry Ford Hospital, Vattikuti Urology Institute, Detroit, MI USA; 5 Department of Urology, IRCCS Regina Elena National Cancer Institute, Rome, Italy; 6 Department of Urology, Fox Chase Cancer Center, Philadelphia, PA USA; 7 Division of Urology, European Institute of Oncology IRCCS, Milan, Italy; 8 Division of Urology, University of Turin San Luigi Gonzaga Hospital, Turin, Italy; 9 Department of Surgery, Candiolo Cancer Institute, FPO-IRCCS, Candiolo, Turin, Italy; 10 Department of Urology, Istituto Nazionale Tumori IRCCS Fondazione G. Pascale, Naples, Italy; 11 Department of Urology, University of Texas Southwestern Medical Center, Dallas, TX USA; 12 John Hopkins University, The James Buchanan Brady Urological Institute, Baltimore, MD USA; 13 Swedish Medical Center, Seattle, WA USA; 14 Section of Urologic Oncology, Rutgers Cancer Institute and Rutgers Robert Wood Johnson Medical School, New Brunswick, NJ USA; 15 Oncologic Minimally Invasive Urology and Andrology Unit, Careggi Hospital, University of Florence, Florence, Italy; 16 Department of Urology, Duke University, Durham, NC USA; 17 University of Miami Miller School of Medicine, Desai Sethi Urology Institute, Miami, FL USA; 18 Department of Urology, Indiana University, Indianapolis, IN USA; 19 Department of Urology, Icahn School of Medicine at Mount Sinai Hospital, New York, NY USA; 20 Kansai Medical University, Osaka, Japan; 21 Department of Urology, University of Alabama at Birmingham, Heersink School of Medicine, Birmingham, AL USA; 22 Department of Urology, UC San Diego School of Medicine, La Jolla, San Diego, CA USA; 23 Norris Comprehensive Cancer Center, Institute of Urology, University of Southern California, Los Angeles, CA USA

**Keywords:** Postoperative complications, Radical nephroureterectomy, Renal insufficiency, Solitary kidney, Upper tract urothelial carcinoma

## Abstract

**Purpose:**

Radical nephroureterectomy (RNU) for upper tract urothelial carcinoma (UTUC) in solitary kidney patients is a rare and underreported scenario. This study aims to compare the outcomes of UTUC solitary kidney patients becoming anephric after RNU to those of patients undergoing kidney-sparing surgery (KSS).

**Methods:**

Data from patients with a solitary kidney were retrieved from the ROBUUST 2.0 database, a global, multicenter registry containing data on patients who underwent curative surgery for UTUC. Baseline patient demographics, disease characteristics, and surgical features were compared between RNU and KSS. Kaplan-Meier methods were used to estimate recurrence-free survival (RFS), metastasis-free survival (MFS), cancer-specific survival (CSS), and overall survival (OS) in patients undergoing RNU, with 3-year and 5-year cutoffs applied.

**Results:**

Thirty-nine patients (76.5%) underwent RNU, whereas 12 (23.5%) underwent KSS. Despite a comparable preoperative renal function, the distribution of CKD stages differed significantly between the groups (*p* = 0.019). Despite a similar rate of postoperative complications, patients undergoing RNU experienced a significantly higher median LOS (*p* < 0.001). Among RNU patients, OS was 83.9%, CSS was 96.9%, RFS was 71.8%, and MFS was 84.4% at the 3-year follow-up. After 5 years post-surgery, OS was 73.4%, CSS was 83.1%, RFS was 59.9%, and MFS was 78.5% in the same cohort.

**Conclusions:**

UTUC solitary kidney patients undergoing RNU or KSS face a substantial perioperative burden. Despite these challenges, our cohort demonstrated favorable oncological outcomes comparable to those reported in the existing literature.

**Supplementary Information:**

The online version contains supplementary material available at 10.1007/s00345-025-05882-0.

## Introduction

Patients with a solitary kidney represent a unique clinical scenario in which treatment decisions must carefully balance oncological outcomes with the preservation of renal function. KSS is a viable option for this subgroup, even in high-risk patients [1]. Nevertheless, RNU may still be necessary for cases involving tumors not amenable to KSS or in patients with end-stage renal disease (ESRD), for whom the only alternative is often palliative treatment, including urinary diversion. Anephric status profoundly reduces the quality and quantity of life due to lifelong hemodialysis and fluid restrictions [2]. Furthermore, UTUC aggressiveness limits the prospects for renal transplantation, making this approach an exceptional and seldom-adopted option.

The present study reports the outcomes of solitary kidney patients undergoing RNU or KSS using a large multi-institutional dataset, offering insights and evidence to support clinical decision-making in this relatively uncommon scenario.

## Materials and methods

### Study population

The ROBUUST (ROBotic surgery for Upper tract Urothelial cancer STtudy) 2.0 is a global, multicenter registry collecting data on patients undergoing curative surgical interventions—RNU or KSS, including segmental ureterectomy and endoscopic ablation—for UTUC at participating institutions from 2015 to 2024. Ethical approval and data-sharing permissions were granted at all participating centers (*IRB: 22111001*). As per study protocol, patients underwent scheduled follow-up visits with quarterly urinary cytology and cystoscopy for the first two years, along with quarterly CT scans during the first year, followed by annual assessments thereafter. Additionally, endoscopic follow-up for patients undergoing KSS adhered to international guideline recommendations. All surgeries were performed by experienced urological surgeons at tertiary referral centers following standardized techniques. Data retrieval from the ROBUUST database was limited to solitary kidney patients undergoing either RNU or KSS, excluding those with incomplete data on relevant predictors.

### Study variables and outcomes

Baseline demographics and disease features were recorded. Surgical variables included the adopted technique and approach, the execution of a single-stage technique and of a concomitant radical cystectomy, operative time (OT), estimated blood loss (EBL), bladder cuff management, lymph node dissection, type and grade of intraoperative and postoperative complications graded according to the standardized Clavien-Dindo (CD) classification [3] (defined as major when grade was ≥ CD III), and length of stay (LOS). Additionally, a subanalysis by complication type was carried out to clarify the etiology of postoperative complications. Pathological features, such as tumor stage and grade, margin status, nodal stage, and lymph node yield were reported.

Functional data (serum creatinine [SCr] and estimated glomerular filtration rate [eGFR]) were recorded before surgery, at the time of discharge, at 3 and 12 months postoperatively, and at the last recorded follow-up after surgery. The eGFR was calculated using the Chronic Kidney Disease Epidemiology Collaboration 2021 formula [4].

All-cause mortality was defined as death from any cause, while cancer-specific mortality referred to deaths directly attributed to cancer. Oncological time-to-event outcomes included recurrence-free survival (RFS), defined as the time from diagnosis to urothelial recurrence into the bladder or homolateral upper urinary tract; metastasis-free survival (MFS), defined as the time from diagnosis to distant metastasis; cancer-specific survival (CSS), and overall survival (OS), defined as the time from diagnosis to tumor-related death and death from any cause, respectively.

### Statistical analysis

Statistical analyses followed established guidelines [5, 6]. The cohort was divided into two groups according to treatment modality. Continuous variables were reported using median and interquartile ranges (IQR), while categorical variables were summarized using proportions and frequencies. Descriptive statistical analysis was carried out as appropriate. Variation in eGFR was calculated as the difference between the baseline and the last follow-up values. Kaplan-Meier methods were employed to calculate local RFS, distant MFS, CSS, and OS, with a 3-year and 5-year cutoff applied. To analyze oncological outcomes based on renal function and surgical approach (RNU vs. KSS), preplanned subanalyses stratifying survival data by chronic kidney disease (CKD) stage and treatment type were conducted, using the log-rank test to assess statistical differences between subgroups. Follow-up duration was calculated from treatment to recurrence or metastasis for RFS and MFS, and from treatment to death or last follow-up for CSS and OS. Statistical analyses were conducted using Stata^®^ v17.0 (StataCorp LLC, College Station, TX, USA), with statistical significance set at *p* < 0.05.

## Results

### Baseline features

In the overall cohort, 3,216 patients underwent surgical treatment for UTUC and were included in the dataset, of whom 51 (1.6%) had a solitary kidney before intervention. Among them, 39 (76.5%) underwent RNU, whereas the remaining patients (12, 23.5%) underwent KSS, including 10 segmental ureterectomies and two endoscopic ablations, with a median follow-up of 14 months (5–48). Baseline characteristics are summarized in Table 1. The median (IQR) age of patients undergoing RNU was significantly higher (80 years, 76-81.5), compared to those undergoing KSS (70 years, 66–78, *p* = 0.006). Despite the median preoperative SCr and eGFR being comparable between patients undergoing RNU and KSS, the distribution of CKD stages differed significantly between the groups (*p* = 0.019). However, the remaining baseline features were comparable between the two subgroups. Namely, in the RNU subgroup, the renal pelvis (21, 53.9%) was the most common tumor site, compared to the ureteral tumors in the KSS subgroup (9, 75%), with no significant difference in terms of tumour site (*p* = 0.051).

**Table 1:** Baseline features of solitary kidney UTUC patients undergoing RNU vs. KSS

Variables	RNU (*n* = 39)	KSS* *(n = 12)*	*P* value
Age, median (IQR)	80 years (76-81.5)	70 years (66–78)	**0.006**
Sex, n (%)			
Male	31 (79.5)	8 (66.7)	0.442
Ethnicity, n (%)			
Caucasian	20 (51.3)	9 (75)	0.220
Black	2 (5.1)		
Hispanic	2 (5.1)	1 (8.33)	
Asian	14 (35.9)	1 (8.33)	
Other	1 (2.6)	1 (8.33)	
Smoke, n (%)			
No	22 (56.4)	3 (25)	0.067
Former	11 (28.2)	8 (66.7)	
BMI, median (IQR)	24.1 kg/m^2^ (22.6–30.1)	26.1 kg/m^2^ (23-31.1)	0.562
Hypertension, n (%)	22 (56.4)	10 (83.3)	0.171
Diabetes mellitus, n (%)	12 (30.8)	3 (25)	0.503
History of BC, n (%)			
Low grade	10/37 (27)	0 (0)	0.09
High grade	7/37 (18.9)	3 (25)	
Previous BC surgery, n (%)			
TURB-T	14/37 (37.8)	3 (25)	1
Radical cystectomy	3 (8.1)	0	
Symptoms, n (%)			
Hematuria	21 (53.8)	7 (58.4)	0.227
Flank pain	2 (5.1)	0 (0)	
Asymptomatic	7 (18)	4 (33.3)	
Other/Unknown	9 (23.1)	1 (8.3)	
Preop Cr, median (IQR)	1.45 mg/dL (1.06–2.16)	1.37 mg/dL (1.12–1.54)	0.377
Preop eGFR, median (IQR)	45.5 mL/min/1.73m^2^ (28–56)	47 mL/min/1.73m^2^ (43.9–59.5)	0.434
CKD stage, n (%)			
0	4 (10.3)	1 (8.3)	**0.019**
I	5 (12.8)	3 (25)	
II	6 (15.4)	1 (8.3)	
III	14 (35.9)	7 (58.4)	
IV	5 (12.8)	0 (0)	
V	5 (12.8)	0 (0)	
Tumor size, median (IQR)	2.5 cm (2–4)	1.9 cm (1.5-3)	0.172
Tumor site, n (%)			
Pelvis	21 (53.9)	1 (8.3)	0.051
Calyx	3 (7.7)	1 (8.3)	
UPJ	2 (5.1)	1 (8.4)	
Ureter	11 (28.2)	9 (75)	
Kidney and ureter	2 (5.1)	0 (0)	
Multifocal, n (%)	10 (25.6)	6 (50)	0.157
Hydronephrosis, n (%)	18 (46.1)	4 (33.3)	0.511
Tumor stage, n (%)			
cTa	8 (20.5)	9 (75)	**0.017**
cT1	14 (36)	1 (8.3)	
cT2	7 (17.9)	1 (8.4)	
cT3-4	7 (17.9)	0 (0)	
Unknown	3 (7.7)	1 (8.3)	
Nodal stage, n (%)			
cN0	37 (94.8)	12 (100)	1
cN1	1 (2.6)	0 (0)	
cN2	1 (2.6)	0 (0)	
Metastatic disease, n (%)	1 (2.6)	1 (8.3)	0.434
Preoperative URS, n (%)	23 (59)	9 (75)	0.494
Biopsy grade, n (%)			
Low grade	4/23 (17.4)	3/9 (33.3)	0.661
High grade	13/23 (56.5)	6/9 (66.7)	
Risk stratification			
EAU High risk	37 (94.9)	11 (91.7)	0.561
NCCN High risk	34 (87.2)	12 (100)	0.323
Neoadjuvant therapy, n (%)	9 (23.1)	0 (0)	0.094

*P*-values for statistically significant results are highlighted in bold

*BC* bladder cancer; *BMI* body mass index; *CKD* chronic kidney disease; *Cr* serum creatinine; *eGFR* estimated glomerular filtration rate; *KSS* kidney sparing surgery; *Preop* preoperative; *TURB* transurethral resection of bladder tumor; *RNU* radical nephroureterectomy; *URS* ureteroscopy; *UTUC* upper tract urothelial carcinoma

*KSS included 10 segmental ureterectomies and two endoscopic ablations

**Table 2:** Postoperative outcomes of solitary kidney UTUC patients undergoing RNU vs. KSS

Variables	RNU (*n* = 39)	KSS* *(n = 12)*	*P* value
Surgical technique, n (%)			0.460
Robotic	21 (53.9)	5/10 (50)	
Laparoscopic	10 (25.6)	1/10 (10)	
Open	8 (20.5)	4/10 (40)	
Approach, n (%)			0.660
Transperitoneal	31 (79.5)	9/10 (90)	
Single stage procedure, n (%)	22 (56.4)		
Concomitant radical cystectomy, n (%)	12 (30.8)	1 (10)	0.252
OT, median (IQR)	244.5 min (165.5-353.5)	162.5 min (109.5–270)	0.102
EBL, median (IQR)	200 mL (100–366)	100 mL (50–100)	**0.013**
Bladder cuff Management, n (%)			0.246
Open extravesical excision	5/35 (14.3)	3/10 (30)	
Minimally invasive transvesical	2/35 (5.7)	1/10 (10)	
Minimally invasive extravesical	17/35 (48.5)	5/10 (50)	
Pluck technique	1/35 (2.9)	1/10 (10)	
Unroofing	1/35 (2.9)	0 (0)	
Not removed	9/35 (25.7)	0 (0)	
Lymph node dissection, n (%)	20 (51.3)	5/10 (50)	1
Intraoperative complications	2 (5.3)	1 (8.3)	1
30-day postop complications			
Yes	14 (35.9)	5 (41.7)	1
CD ≥ 3	8 (20.5)	1 (8.3)	0.666
Readmission	4/36 (11.1)	3/11 (27.3)	0.330
LOS, median (IQR)	7.5 days (5–12)	3 days (2–5)	**< 0.001**
Pathological stage, n (%)			0.429
pTa	9 (23.1)	4 (33.3)	
pTis	2 (5.1)	0 (0)	
pT1	6 (15.4)	3 (25)	
pT2	5 (12.8)	2 (16.7)	
pT3-4	17 (43.6)	2 (16.7)	
Tumor Grade, n (%)			0.133
High grade	36 (92.3)	9 (75)	
Positive Margins, n (%)	1 (2.6)	3 (25)	**0.038**
pN			0.156
pN0	23 (59)	3 (25)	
≥pN1	2 (5.1)	2 (16.7)	
pNx	14 (35.9)	7 (58.3)	
Adjuvant treatments			
Bladder instillation	4 (10.3)	2 (16.7)	0.616
Systemic therapy	2/36 (5.6)	2 (16.7)	0.257
Postoperative Cr			
Discharge	6 mg/dL (3.45–7.81)	1.3 mg/dL (1.2–1.6)	**< 0.001**
12 months	4.1 mg/dL (1.48–7.5)	1.5 mg/dL (1.4–1.6)	0.074
Last follow-up	5.9 mg/dL (2-8.8)	1.5 mg/dL (0.75–1.8)	**0.009**
Postoperative eGFR			
Discharge	9 mL/min/1.73m^2^ (6.8–15.1)	50 mL/min/1.73m^2^ (30–53)	**< 0.001**
12 months	12.2 mL/min/1.73m^2^ (6–44)	46 mL/min/1.73m^2^ (32–48)	0.111
Last follow-up	7.5 mL/min/1.73m^2^ (6–32)	38 mL/min/1.73m^2^ (32–85)	**0.012**
ΔeGFR	-18.1 mL/min/1.73m^2^ (-34;1)	-2 mL/min/1.73m^2^ (-5;21)	0.101

*P*-values for statistically significant results are highlighted in bold

*CD* Clavien-Dindo; *Cr* serum creatinine; *EBL* estimated blood loss; *eGFR* estimated glomerular filtration rate; *KSS* kidney sparing surgery; *LOS* length of stay; *OT* operative time; *Postop* postoperative; *RNU* radical nephroureterectomy; *UTUC* upper tract urothelial carcinoma

*KSS included 10 segmental ureterectomies and two endoscopic ablations

### Perioperative outcomes

Perioperative outcomes are reported in Table 2. Robotic surgery was the most common approach (21 RNU, 53.9% vs. 5/10 KSS, 50%; *p* = 0.460), regardless of the surgery type. A median operative time of 244.5 min (IQR: 165.5–353.5), and a median EBL of 200 mL (100–366) was registered for RNU, compared to a median operative time of 162.5 min (109.5–270, *p* = 0.102), and a median EBL of 100 mL (50–100, *p* = 0.013) when looking at KSS. Median LOS was statistically significantly longer for RNU (7.5 days, 5–12) than KSS (3 days, 2–5; *p* < 0.001). Moreover, similar rates of any grade (RNU: 35.9% vs. KSS: 41.7%, *p* = 1) and major (RNU: 20.5% vs. KSS: 8.3%, *p* = 0.666) postoperative complications were observed among the two subgroups. Postoperative acute kidney injury occurred in 39 RNU patients (100%) and 2 KSS patients (16.7%), with no significant difference in terms of complications type between the subgroups (*p* = 0.241), as depicted in Supplementary Table 1. Additionally, the analysis of postoperative functional outcomes revealed significantly higher SCr and lower eGFR at both discharge and last follow-up in patients undergoing RNU compared to KSS (all *p* < 0.05).

### Pathological outcomes

Among patients undergoing RNU, more than half (22, 56.4%) had muscle-invasive disease (≥ pT2) on final histology, with the majority being high grade (36, 92.3%). Positive surgical margins were observed in only one patient (2.6%), and nodal involvement in two patients (5.1%). In contrast, most patients undergoing KSS had non–muscle-invasive tumors (7, 58.3%; *p* = 0.429) but exhibited a significantly higher rate of positive surgical margins (3, 25%; *p* = 0.038) and a comparable rate of nodal involvement (2, 16.7%; *p* = 0.156).

### Survival analysis

In the RNU cohort, the recurrence rate was 20.5%, while all-cause mortality and cancer-specific mortality were 12.8% and 5.1%, respectively. On the other hand, the recurrence rate was 25% (*p* = 0.584) among patients undergoing KSS, including two bladder recurrences and one renal pelvis recurrence, with all-cause mortality and cancer-specific mortality rates of 25% (*p* = 0.379) and 8.3% (*p* = 1), respectively. The intended subanalyses by CKD stage and treatment type were not feasible due to the low number of events observed in each group. Therefore, survival analysis was conducted exclusively on patients undergoing RNU. At the 3-year follow-up, OS was 83.9%, CSS was 96.9%, RFS was 71.8%, and MFS was 84.4%. After 5 years post-surgery, OS was 73.4%, CSS was 83.1%, RFS was 59.9%, and MFS was 78.5%.

## Discussion

This study is the first to compare outcomes of solitary kidney UTUC patients undergoing RNU, resulting in anephric status, and KSS in a multicenter international setting. Despite the postoperative functional challenges faced by patients undergoing RNU, our findings indicate that their oncological outcomes are comparable to those reported in the existing literature. However, these results come at the cost of a higher perioperative burden, as evidenced by prolonged LOS.

Opting for RNU in solitary kidney patients is an uncommon decision, as it inevitably leads to dialysis dependence, significantly impacting patients’ quality of life. This challenge likely accounts for the paucity of data in this clinical setting, limited to case reports and small case series [7, 8]. In contrast, the ROBUUST registry includes data on over 2,900 patients, allowing us to analyze the largest available sample size of solitary kidney patients undergoing RNU to date. Most patients in our cohort were classified as high-risk, making them eligible for KSS due to imperative indications despite their risk classification [1]. However, the decision to perform RNU in such cases may have been driven by severe preoperative renal function impairment, irrespective of risk classification. Indeed, in our cohort, 25% of patients had a preoperative eGFR below 30 mL/min/1.73m^2^, while none of the KSS patients did, possibly explaining the preference for extirpative surgery in the former subgroup. When looking at the role of preoperative renal function in guiding surgical decision-making for solitary kidney patients with UTUC, a single-center retrospective study by Su et al. provides valuable insights on this topic. In this cohort, over half of the patients had a history of aristolochic acid exposure, which contributed to a high incidence of synchronous (3.5%) and metachronous (4.8%) bilateral UTUC. Among the patients evaluated, 29 (47.5%) underwent RNU, while 32 (52.5%) received KSS. The findings demonstrated a significant association between preoperative renal function and the choice of surgical intervention, as patients undergoing RNU had significantly worse baseline eGFR than those receiving KSS [8].

The role of preoperative renal function in UTUC patients extends beyond influencing surgical treatment choice, potentially contributing to the risk of occurrence/recurrence of UTUC in solitary kidney patients. According to Wu et al., patients with ESRD on dialysis exhibited a higher incidence of UTUC along with a greater incidence of synchronous multifocal and bilateral UTUC compared to the general population [9]. Additionally, a population-based retrospective study by Kang et al. found that patients with preoperative SCr levels above 1.4 mg/dL had more than a threefold increased risk of contralateral recurrence [10]. Consistent with these results, laboratory findings indicated suboptimal renal function in both the study cohorts, potentially identifying a group of patients at increased risk for either primary UTUC in a solitary kidney or contralateral/homolateral UTUC recurrence. The field cancerization hypothesis provides the most plausible explanation for the increased risk of UTUC development in this cohort, suggesting that the urothelium is uniformly predisposed to malignant transformation due to shared exposure to carcinogens [11]. Indeed, nephrotoxic and carcinogenic agents have been implicated in both the progression of renal disease and the neoplastic transformation of urothelial tissue [10]. Therefore, prior exposure to these nephrotoxic agents by patients with UTUC and a solitary kidney, often linked to conditions such as ESRD and prolonged dialysis, could further exacerbate this risk.

From a surgical standpoint, KSS performed via an endoscopic approach can present technical and oncological challenges [12] as well as a relevant risk of stenotic sequelae [13]. Similarly, complex cases (i.e. large endoluminal defects) treated with renal or pelvic resection or segmental/total ureterectomy can be highly demanding and carry a significant risk of tumor seeding due to urine spillage [14]. Notably, although the difference in tumor site between the two cohorts only approached statistical significance (*p* = 0.051), with three-fourths of KSS patients having ureteral tumors compared to only one-fourth in the RNU group, a potential clinical significance can be speculated. Thus, despite growing evidence supporting the feasibility of ureterectomy in high-risk patients [15–17] certain extreme scenarios necessitate RNU, even in solitary kidney patients.

What stands out from our analysis is the high incidence of any grade and major postoperative complications, regardless of the treatment type. Among patients undergoing RNU, 35% experienced any-grade postoperative complications, while 20.5% had major complications. Conversely, previous analyses of the ROBUUST registry reported any-grade complication rates around 15%, with major complications occurring in fewer than 5% of cases [18, 19]. Moreover, a large population-based study by Franco et al. reported an incidence of 30-day postoperative complications of around 20% for RNU [20]. With regards to KSS, existing literature primarily focuses on oncological outcomes, with postoperative complications largely underreported. However, a prior ROBUUST registry analysis recorded any-grade and major complication rates of 15% and 5%, respectively [16]. In comparison, the current study group showed rates of 41.7% and 8.3%, respectively.

Notably, RNU patients in this study had a significantly longer LOS compared to those undergoing KSS. Moreover, when compared with available literature, RNU patients in our cohort demonstrated a prolonged LOS [20, 21] whereas LOS for KSS patients remained consistent with previously reported data [17]. The need for postoperative hemodialysis may partially explain the delayed discharge in the RNU subgroup. As anticipated, most of the postoperative events among patients undergoing RNU were reported as acute kidney injury (39, 100%) following kidney removal, whereas only two events were reported in patients undergoing KSS. These findings highlight the need for thorough preoperative counseling to set realistic expectations regarding complications and recovery. They also emphasize the heightened vulnerability of solitary kidney patients to postoperative renal insults, underscoring the importance of close monitoring and early intervention [22, 23]. Thus, effective hospital management should integrate nephrology collaboration to ensure timely interventions, including dialysis when necessary, to mitigate acute renal dysfunction and long-term sequelae [24].

On the whole, oncological safety and technical feasibility should remain the primary considerations when determining the appropriate therapeutic strategy. In our cohort, patients achieved a 3-year and 5-year OS of 83.9% and 73.4%, respectively, alongside a CSS of 96.9% and 83.1% at the same time points. Survival outcomes following RNU show significant variability in the literature [21, 25, 26] heavily influenced by tumor grade and stage [27] but in line with those observed in our analysis. The concept of surgically induced CKD, first described by Lane et al. [28] refers to the adverse effects of nephron loss following kidney surgery [29]. Its association with increased mortality risk and adverse metabolic effects has been well-documented in renal cell carcinoma but remains underreported in UTUC [30, 31]. In this context, a relationship between survival and renal function was initially proposed by Kim et al. [31] and subsequently corroborated by Puri et al., who reported a progressively declining 5-year OS with worsening postoperative renal function and a significantly higher risk of all-cause mortality [32]. However, a retrospective study by Su et al. comparing 29 with 32 solitary kidney patients undergoing either RNU or KSS for UTUC, respectively, reported similar oncological outcomes to our findings, with no statistically significant difference between treatment arms, and almost 10% of patients undergoing KSS ultimately requiring dialytic treatment [8]. These observations underscore the complex interplay between renal damage and survival outcomes. However, no study to date has specifically examined solitary kidney patients made anephric after surgery, making direct comparisons with our findings both challenging and potentially inconclusive. On one hand, this association could be mitigated in patients receiving dialytic treatment, given the complete replacement of renal function. On the other hand, it is possible that sacrificing renal function in favor of more radical surgery may lead to reduced cancer-specific mortality in this patient group. However, the strength of our findings is likely influenced by the small sample size and the low number of observed events. In addition, we were unable to perform survival subanalyses either based on preoperative renal function or surgical intervention due to the limited number of observations. Furthermore, a causal relationship between mortality and renal function should not be assumed, as the observational design of the studies does not allow for definitive conclusions. Given the limited literature available, dedicated studies are needed to clarify the long-term oncological outcomes and to provide evidence-based guidance on optimal treatment strategies for this cohort.

This study benefits from the use of the ROBUUST 2.0 registry, a large, multi-institutional database, enhancing the generalizability of the findings. By focusing on solitary kidney patients, the present analysis sought to address an underexplored population, helping to fill a gap in the existing literature. However, several limitations must be acknowledged. Due to the absence of this data in the dataset, we were unable to determine the indication for surgery in the included patients, as well as the reason for having a solitary kidney. Moreover, the relatively short median follow-up limited the assessment of long-term oncological outcomes and dialysis-related complications, which may have influenced cancer-specific and all-cause mortality, respectively. Additionally, the small sample size, although relatively large if considering solitary kidney patients undergoing RNU, remains modest compared to other more common clinical populations, potentially reducing the power to detect smaller but clinically meaningful differences. In addition, the number of patients undergoing KSS in our cohort prevented us from performing a dedicated subanalysis by treatment type. The study’s reliance on registry data, together with its retrospective design, introduces potential selection bias, as patients included may differ systematically from those not captured. Finally, variations in follow-up protocols could exist due to the multi-institutional nature of the dataset.

## Conclusion

Solitary kidney patients are a vulnerable group of patients requiring careful consideration of the balance between risks and benefits in the decision-making process. These patients may experience a higher incidence of UTUC compared to the general population. Furthermore, they face a substantial perioperative burden, including high complication rates and prolonged LOS. Despite these challenges, patients undergoing RNU demonstrated favorable oncological outcomes comparable to those reported in the existing literature.

## Supplementary Information

Below is the link to the electronic supplementary material.

Supplementary Material 1

## Data Availability

The data sets generated during and/or analyzed during the current study are available from the corresponding author upon reasonable request.
