# Supplementary material for: Radical nephroureterectomy vs kidney sparing surgery for upper tract urothelial carcinoma in solitary kidney patients: a multi-institutional analysis of the ROBUUST 2.0 registry
Source: World J Urol. 2025 Sep 3;43(1):534. doi: 10.1007/s00345-025-05882-0 (PMC12405301; doi:10.1007/s00345-025-05882-0)
Supplement: Supplementary file 1 — Supplementary Material 1 [file 345_2025_5882_MOESM1_ESM.docx]

**Supplementary Table 1.** Complication number and type

|  | Number (%) | | p value |
| --- | --- | --- | --- |
| Complications | **RNU (n=39)** | **KSS (n=12)** | 0.241 |
| *Acute kidney injury* | 39 (100) | 2 (16.7) |  |
| *Chylus leak* | 2 (14.3) | 0 (0) |  |
| *Ileus* | 2 (14.3) | 1 (8.3) |  |
| *Anemia/Bleeding* | 2 (14.3) | 1 (8.3) |  |
| *Abdominal abscess* | 1 (7.1) | 0 (0) |  |
| *Sepsis* | 1 (7.1) | 1 (8.3) |  |
| *Urinary leakage* | 0 (0) | 1 (8.3) |  |
